# Supplementary material for: Functional Studies of Deafness-Associated Pendrin and Prestin Variants
Source: Int J Mol Sci. 2024 Feb 27;25(5):2759. doi: 10.3390/ijms25052759 (PMC10931795; doi:10.3390/ijms25052759)
Supplement: Supplementary file 1 [file ijms-25-02759-s001.zip › ijms-2874911-supplementary.pdf]

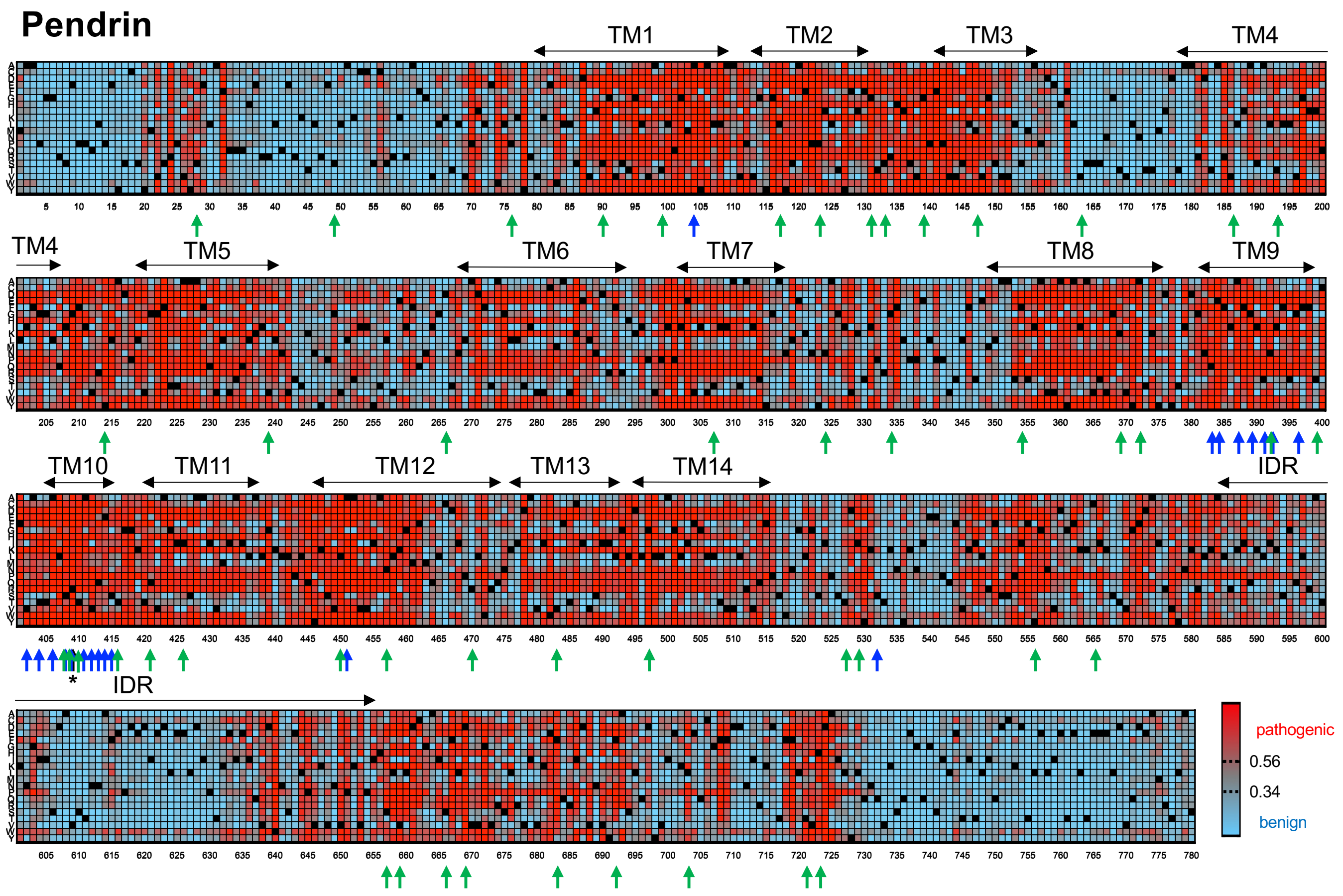


**Supplementary Figure S1. AlphaMissense pathogenicity scores of pendrin.** Heatmap showing the AM scores for the entire length of human pendrin. Variants with a score from 0-0.34 are classified as benign (cyan), 0.34-0.56 as ambiguous (gray), and 0.56-1.0 are pathogenic (red) by AM. Amino acid residue numbers are shown on the bottom and the amino acids (one letter code) are indicated on the left. WT residues are shown in black. Upward arrows indicate the positions of the residues with the variants tested in the previous study (green, [10] or in this study (blue). Black upward arrow with an asterisk indicates the anion binding site. Horizontal double arrows show the regions that comprise the transmembrane (TM) helices and the intrinsically disordered region (IDR).

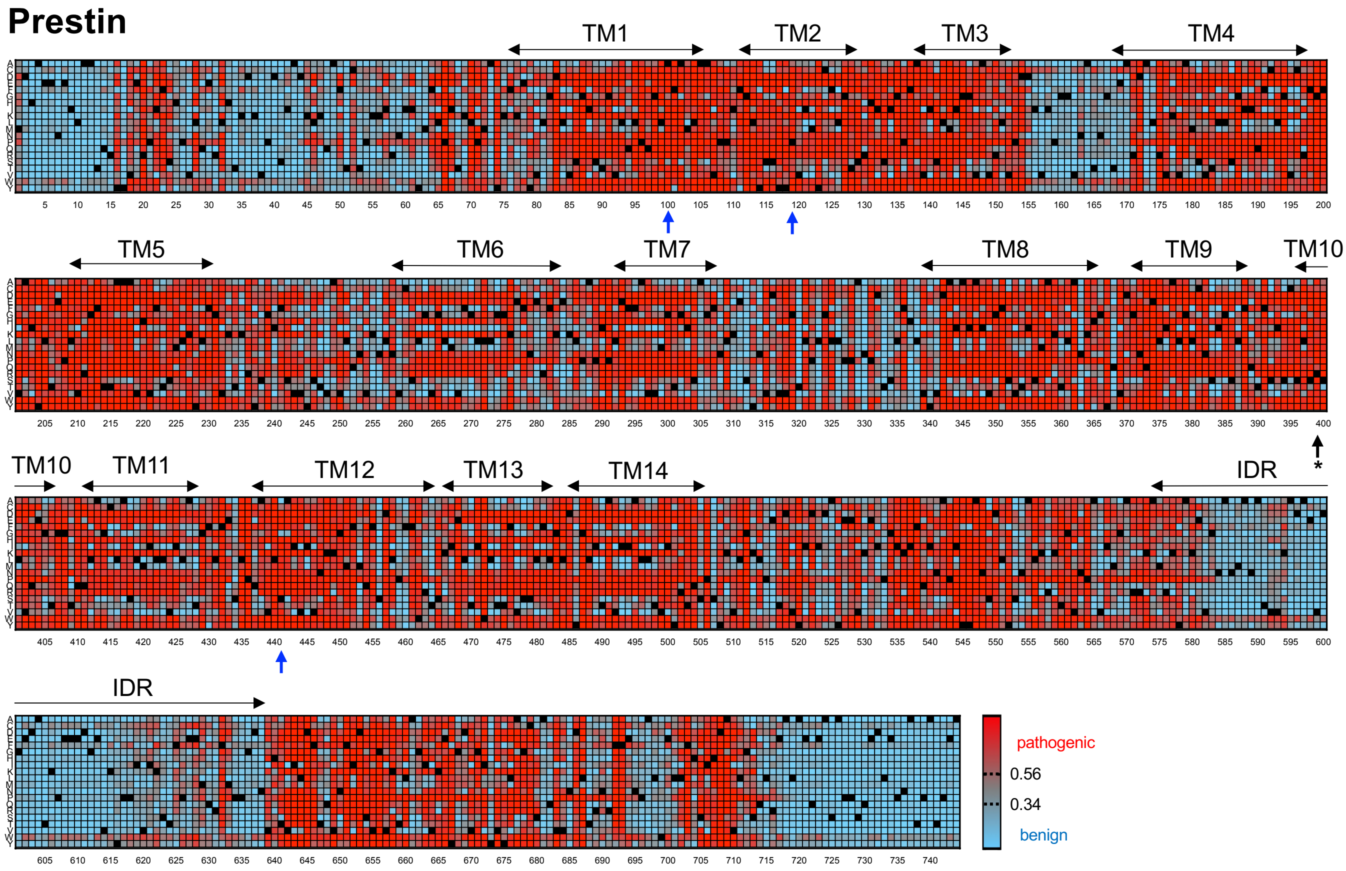


**Supplementary Figure S2. AlphaMissense pathogenicity scores of prestin.** Heatmap showing the AM scores for the entire length of human prestin. AM pathogenicity scores are colored as in Supplemental Figure S1. Amino acid residue numbers are shown on the bottom and the amino acids (one letter code) are indicated on the left. WT residues are shown in black. Blue upward arrows indicate the positions of the residues with variants tested in this study. Black upward arrow with an asterisk indicates the anion binding site. Horizontal double arrows show the regions that comprise the transmembrane (TM) helices and the intrinsically disordered region (IDR).
